# Editorial: Systems and network approaches to precision medicine and healthcare

**DOI:** 10.3389/fmolb.2024.1463962

**Published:** 2024-08-09

**Authors:** Emma L. Kurnat-Thoma, Cristian Nogales, Sona Vasudevan

**Affiliations:** ^1^ Georgetown Institute for Women, Peace and Security, Walsh School of Foreign Service, Georgetown University, Washington, DC, United States; ^2^ Precision Policy Solutions, LLC, Bethesda, MD, United States; ^3^ Department of Structural and Computational Biology, Center for Molecular Biology, University of Vienna, Vienna, Austria; ^4^ Max Perutz Labs, Vienna Biocenter Campus (VBC), Vienna, Austria; ^5^ Department of Biochemistry, Molecular and Cellular Biology, Georgetown University Medical Center, Washington, DC, United States

**Keywords:** precision medicine, systems medicine, network medicine, artificial intelligence and machine learning (AI/ML), healthcare

## Introduction

The one-size-fits-all approach in medicine does not work (Nogales et al., 2022). Complex multifactorial disease mechanisms involve multiple dysfunctional signaling networks, regulatory components, organs, and systems factors. Although precision medicine (PM) targets causal monogenic disease mechanisms and promotes curative amelioration, network and systems science is required to avoid piecemeal assembly of single molecules (Kurnat-Thoma, 2020; Kurnat-Thoma et al., 2020).

**FIGURE 1:**
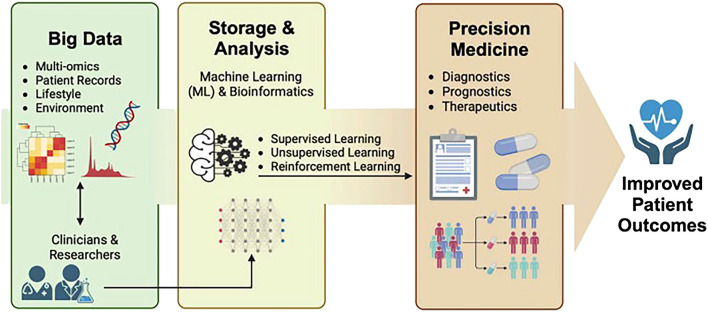
Systems and network approaches use big data and machine learning to obtain improved precision medicine patient outcomes when studying the human gastrointestinal microbiome (Wu et al., 2024).

The “*systems and network approaches to precision medicine and healthcare”* Research Topic brings together various interdisciplinary systems and network medicine approaches to highlight how PM clinical translation is strengthened, patient benefits are increased, and serious harms are reduced (see Figure 1). Finally accepted articles featured a wide diversity of article types, qualitative and quantitative methodologies, medical specialties, and advanced technological, regulatory, and policy considerations.

### Pro-Cure PM therapeutics database

Clinical use of off-label pharmacological therapeutics for hard-to-treat cancers in pediatric oncology is understudied and underdeveloped (Lim et al., 2020). Mazariego et al. performed an original qualitative research study to design Pro-Cure, a novel pediatric oncology PM database. Using a reputable implementation science framework, study investigators interviewed 17 multidisciplinary pediatric oncology healthcare professionals to understand end-user preferences and needs. They collaboratively engineered a process map and web interface to facilitate Pro-Cure’s platform acceptability. The ProCure beta resource allowed providers to search for a drug and company given Multidisciplinary Tumor Board recommendations on a tumor’s molecular profile (i.e., *EGFR, KIT, RET, VEGFR2)*. It also supported evidence reviews, regulatory and pharmaceutical company documentation in a pediatric oncology network. ProCure significantly decreased provider and consultant workloads and facilitated access to off-label therapeutics for a wide range of hard-to-treat cancers in vulnerable children and their families.

### Clinical prediction nomograms

Nomograms are multi-metric models that can evaluate and predict cancer survival. The retrospective analysis performed by Li et al. identified independent predictive factors for cancer-specific survival (CSS) of malignant adrenal tumors. Their team analyzed National Cancer Institute (NCI) Surveillance, Epidemiology, and End Results (SEER) data from 1,748 patients with malignant adrenal tumor diagnoses from 2000–2019. Age, tumor stage, size, histological grade and treatment types, were used in univariate and multivariate Cox regression analyses to develop predictive 3-, 5-, and 10-year CSS nomograms. Robustness was validated through calibration curves, receiver operating characteristic (ROC) curves, and decision curve analysis (DCA), demonstrating high discriminative power and clinical relevance. This study presents a new method of individualized diagnostic risk categorization based on nomogram values, enabling improved treatments and clinical decision-making.

### Improved treatment accuracy via ETU-Net model

Epistaxis is a common emergency department otolaryngology presentation, but effective evaluation and management are limited by inexperienced front line personnel. The original research by Chen et al. used AI/ML applications to enhance endoscopic imaging predictive capacity and reduce severe epistaxis complications. The team assembled a Nasal Bleeding dataset with senior clinicians for image segmentation model learning, proposed the ETU-Net model, which combined convolutional neural network (CNN) supervised learning and Transformer deep learning architecture for segmentation tasks, and compiled several dataset models for comparative performance assessment and advanced model training and testing. The novel ETU-Net model with CNN and Transformer deep learning architecture demonstrated superior capability in assisting physicians to accurately identify ambiguous, extremely fine bleeding areas and abnormal vasculature in endoscopic imaging.

### ML hematological PM applications

Recent ML advancements are transforming the manual standard of bone marrow cell morphology clinical diagnostics, analysis and evaluation. A review by Lin et al. explores how the latest ML algorithms and techniques are innovating identification of hematological disorders such as acute lymphoblastic leukemia (ALL). Their multidimensional focus identifies how automated ML technologies enhance the accuracy, efficiency, and reliability of analyzing the morphology of bone marrow cell samples. They highlight how automated image analysis, improved classification accuracy, and integrating advanced point-of-care capabilities with microfluidics and multimodal imaging into clinical workflows hold great promise for earlier detection of cytopathic changes and improved patient treatment planning.

### ML Multi-omics approaches in gastrointestinal microbiome

The gastrointestinal microbiome plays a critical role in human health, influencing numerous physiological processes and disease outcomes. The mini-review by Wu et al. highlights impacts of high-throughput sequencing technologies as well as the utility of big data approaches for -omics and ML for enhanced biomarker predictive capacity. ML approaches efficiently identify functional genes, microbial compositions, and healthy- and disease-relevant metabolic profiles for potential therapeutic targets that enhance PM capabilities. Future research is needed to fully address and improve patient outcomes through accurate personalized diagnostics, prognostics, and therapeutics in clinical settings.

### PM advances in gestational diabetes mellitus (GDM)

The mini-review by Biete and Vasudevan examines the crucial role of gut microbiota dysbiosis in GDM, and identifies the interconnectedness between maternal and fetal microbiomes in fetal neurodevelopment. They demonstrate how a systems medicine approach can use this information to design personalized patient treatment plans based on microbial abundance levels, microbial metabolites in the maternal gut microbiome, and cord blood to supplement the nutrients needed for fetal neurodevelopment in GDM pregnancies. The significance of leveraging advanced technologies for early disease detection and the importance of managing conditions like GDM for better health outcomes through meaningful insights from large datasets is highlighted. Integrating automated diagnostic tools with comprehensive GDM management strategies can lead to more effective and personalized healthcare solutions.

### Safe and high quality laboratory developed tests (LDTs)

A critical requirement for implementing PM’s promise of the “right treatment, for the right person, and at the right time” in the AI/ML era, is ensuring molecular diagnostic accuracy. However, the vision of rapid clinical translation and implementation of genomic sequencing into routine practice is increasingly impacted by regional health system capacity, infrastructure variability, regulatory approval considerations, reimbursement limitations, and workforce deficiencies (Marshall et al., 2024). A policy brief by Kurnat-Thoma presents policy analysis results and three policy reform strategy recommendations for strengthened regulatory compliance oversight, ensuring PM healthcare quality and patient safety for LDT molecular diagnostics in systems and network medicine.

## Dual use research of concern (DURC) risks

Big data and AI/ML innovations, particularly CRISPR genome editing techniques, synthetic biology and bioengineered devices, are being perpetuated, amplified, and distributed at system scale across the U.S. and globally. The perspective piece by DiEuliis and Giordano summarizes the serious consequences and risks of failing to responsibly wield advanced genomic scientific technologies and capabilities, including intentional misuse by bad actors to incur harm. DURC is the study of biological and life science methods, materials, or results that could be directly misused or weaponized with widescale public health impacts (National Institutes of Health, 2023; Executive Office of the President of the United States, 2024). DURC biotechnology hazards have increasing stewardship implications, including cyberbiosecurity, an emerging novel area of science governance and national security policy (George, 2019). Considerations for mitigating biotechnology DURC contingencies, risks, and threats to ensure ethical PM are reviewed, and future international policy directions are opined.

## Conclusion

This Research Topic highlights the transformative potential of developing and integrating innovative systems and network medicine approaches within healthcare. The published articles provide a comprehensive PM perspective and demonstrate how crucial it is to move from a one-size-fits-all paradigm and use systems and network medicine to improve personalized disease diagnosis, prognosis, and therapeutic options.
